# Evidence That Calcium Entry Into Calcium-Transporting Dental Enamel Cells Is Regulated by Cholecystokinin, Acetylcholine and ATP

**DOI:** 10.3389/fphys.2018.00801

**Published:** 2018-07-02

**Authors:** Meerim K. Nurbaeva, Miriam Eckstein, Arun Devotta, Jean-Pierre Saint-Jeannet, David I. Yule, Michael J. Hubbard, Rodrigo S. Lacruz

**Affiliations:** ^1^Department of Basic Science and Craniofacial Biology, New York University College of Dentistry, New York, NY, United States; ^2^Department of Pharmacology and Physiology, University of Rochester, Rochester, NY, United States; ^3^Faculty of Medicine, Dentistry and Health Sciences, The University of Melbourne, Parkville, VIC, Australia

**Keywords:** ameloblasts, Ca^2+^, CRAC channels, SOCE, CCK, ATP, ACh

## Abstract

Dental enamel is formed by specialized epithelial cells which handle large quantities of Ca^2+^ while producing the most highly mineralized tissue. However, the mechanisms used by enamel cells to handle bulk Ca^2+^ safely remain unclear. Our previous work contradicted the dogma that Ca^2+^ is ferried through the cytosol of Ca^2+^-transporting cells and instead suggested an organelle-based route across enamel cells. This new paradigm involves endoplasmic reticulum (ER)-associated Ca^2+^ stores and their concomitant refilling by store-operated Ca^2+^ entry (SOCE) mediated by Ca^2+^ release activated Ca^2+^ (CRAC) channels. Given that Ca^2+^ handling is maximal during the enamel-mineralization stage (maturation), we anticipated that SOCE would also be elevated then. Confirmation was obtained here using single-cell recordings of cytosolic Ca^2+^ concentration ([Ca^2+^]_cyt_) in rat ameloblasts. A candidate SOCE agonist, cholecystokinin (CCK), was found to be upregulated during maturation, with *Cck* transcript abundance reaching 30% of that in brain. CCK-receptor transcripts were also detected and Ca^2+^ imaging showed that CCK stimulation increased [Ca^2+^]_cyt_ in a dose-responsive manner that was sensitive to CRAC-channel inhibitors. Similar effects were observed with two other SOCE activators, acetylcholine and ATP, whose receptors were also found in enamel cells. These results provide the first evidence of a potential regulatory system for SOCE in enamel cells and so strengthen the Ca^2+^ transcytosis paradigm for ER-based transport of bulk Ca^2+^. Our findings also implicate enamel cells as a new physiological target of CCK and raise the possibility of an auto/paracrine system for regulating Ca^2+^ transport.

## Introduction

Dental enamel forms in localized extracellular microenvironments under the tight control of enamel cells (ameloblasts) and requires a substantial supply of Ca^2+^. Enamel formation occurs in two main stages, termed secretory and maturation, with Ca^2+^ transport increasing fourfold during the latter. Determining how these specialized epithelial cells handle and transport Ca^2+^ in bulk while avoiding the toxic effects associated with high cytosolic concentration is an important issue (Hubbard, 2000). Ameloblasts transport Ca^2+^ predominantly via a transcellular (active) route. Commonly in epithelia, such active systems require logical steps of Ca^2+^ entry, transit and extrusion while changes in cytosolic calcium ([Ca^2+^]_cyt_) are retained for cell signaling. In many tissues the safe transit step has been ascribed to the vitamin-D regulated family of cytosolic Ca^2+^-binding proteins known as calbindins (Feher et al., 1992). However, biochemical investigations failed to support such a cytosolic transit route in enamel (Hubbard, 1996; Turnbull et al., 2004; Hubbard et al., 2011). Instead, proteins associated with endoplasmic reticulum (ER) Ca^2+^ handling were found to be increased during enamel maturation (Hubbard et al., 2000; Franklin et al., 2001) leading to a new paradigm for Ca^2+^ transport termed “calcium transcytosis” (Hubbard, 1996, 2000). Our subsequent discovery of store-operated Ca^2+^ entry (SOCE) mediated by the Ca^2+^ release activated Ca^2+^ (CRAC) channels is in agreement with the calcium transcytosis model in enamel cells (Nurbaeva et al., 2015a,b; Eckstein et al., 2017).

Ca^2+^ release activated Ca^2+^ channels mediate Ca^2+^ influx in many cell types (Parekh and Putney, 2005; Prakriya and Lewis, 2015). The main components of the CRAC channel are an ER Ca^2+^ sensor named STIM1, and the pore subunit of the channel known as ORAI1 found in the plasma membrane (Feske et al., 2006; Feske, 2009; Prakriya and Lewis, 2015). CRAC channels are activated following a decrease of [Ca^2+^] in the ER (Prakriya and Lewis, 2015). CRAC channel activation in ameloblasts appears to be mediated by inositol trisphosphate receptors (IP_3_R) because several IP_3_R subtypes were found to be upregulated during maturation (Nurbaeva et al., 2015a). The expression of STIM1 and ORAI1 proteins in rat enamel cells is also increased during maturation (Nurbaeva et al., 2015a). Given the implicit role of SOCE as a key mediator of Ca^2+^ influx, particularly during enamel maturation, it seems likely that SOCE activation will be tightly regulated. A number of physiological agonists are known to induce changes in [Ca^2+^]_cyt_ by acting on cell surface G-protein coupled receptors (GPCRs) that promote IP_3_ production by phospholipase C (Yule et al., 1991; Cancela, 2001; Foskett et al., 2007). The IP_3_ in turn acts on IP_3_R channels found in the ER membrane and so releases Ca^2+^ from ER/Ca^2+^ stores to stimulate SOCE (Yule and Williams, 1994; Foskett et al., 2007; Mikoshiba, 2007; Lur et al., 2011).

In our previous genome-wide screening comparing enamel organ (EO) cells from secretory and maturation stage, we identified cholecystokinin (CCK) as being highly up-regulated in maturation (Lacruz et al., 2012). CCK is best known as a hormone and neuropeptide associated with multiple physiological functions including the release of digestive enzymes from the pancreas, insulin stimulation, cardiovascular function, and serving as an autocrine growth factor (Williams et al., 2002; Carrillo et al., 2007; Rehfeld et al., 2007). Any function of CCK in enamel formation remains unknown but, given its established role as a GPCR agonist involved in Ca^2+^ signaling in pancreatic acinar cells (Yule et al., 1991; Yule and Williams, 1994; Murphy et al., 2008), we hypothesized a link with SOCE activation in ameloblasts. Likewise, we questioned whether two other well-known GPCR agonists, acetylcholine (ACh) and ATP, were involved in Ca^2+^ handling. Our findings support the presence of a SOCE-regulatory mechanism that involves these three GPCR agonists and implicate CCK as a potential regulator of Ca^2+^ transport in enamel cells.

## Materials and Methods

### Animals

All animal procedures were conducted in accordance with the guidelines approved by the Institutional Animal Care and Use Committee (IACUC) of New York University College of Dentistry (protocol # s16-00625).

### Isolation of Enamel Organ Cells and Assessment of Cell Purity

Rats (100–140 gram) were euthanized and mandibles were dissected out. The surrounding soft tissues (i.e., skin, muscle and ligaments) were removed to expose the body of the mandible as described (Lacruz et al., 2012) to be able to delineate the separation between secretory and maturation stages using the molar reference line (Smith and Nanci, 1989). Isolated mandibles were transferred into a plastic dish containing Hanks solution (GIBCO) with 1% Antibiotic-Antimycotic (solution 1) and kept on ice at all times. The isolated cells are referred to as the EO at this stage because it is impossible to differentiate ameloblasts from the surrounding cell types. Isolated secretory and maturation EO cells were collected in Eppendorf tubes containing 1mL of solution 1. Cells were then treated with 1 mg/ml collagenase/dispase (Roche, Tokyo, Japan) in HBSS solution for 20 min at 37°C in a 5%-CO_2_ incubator being manually pipetted every 5 min to mechanically separate the cells. The enzymatic reaction was stopped by adding X-Vivo^TM^ 15 (Lonza, United States) cell media containing 10% FBS and 1% penicillin/streptomycin (solution 2). Cells were centrifuged at 500 ×*g* for 5 min and washed twice with solution 2. Cells were plated on glass cover slips coated with Poly-L-lysine or Cell-Tak (Corning) bathed in solution 2 and maintained overnight at 37°C in a 5%-CO_2_ incubator. Cells were used within 24–48 h after isolation.

The EO is composed largely by ameloblast cells, our main cell of interest. These cells directly orchestrate the development and mineralization of enamel crystals. A source of possible contamination during this procedure is the cells from the connective tissue layer surrounding the EO cells. Previously we have used the FlexStation to obtain averages of [Ca^2+^]_cyt_ measurements of secretory and maturation EO cells (Nurbaeva et al., 2015a). The present study used single cell imaging as this enabled us to increase the purity of the ameloblast population sampled by eliminating potential contamination of fibroblast/connective tissue cells using a fibroblastic marker CD90. To confirm the make-up of the cell population isolated from secretory and maturation EO cells, we used the ameloblast markers amelogenin (AMELX) and ameloblastin (AMBN). Details of these antibodies are shown below.

### Immunofluorescence (IF)

To ensure high ameloblast purity within sampled whole EO cells, we detected ameloblast cells using antibodies against Amelx and Ambn, whereas a contamination of fibroblasts was identified using an anti-CD90. Secretory and maturation stage cells were cultured for 24 h and fixed with 4% paraformaldehyde before incubating with 0.2% TritonX-100 in phosphate-buffered saline (PBS) for 20 min at room temperature. After blocking for 30 min with 2% bovine serum albumin in PBS, sections were incubated overnight at 4°C with appropriate antibodies (anti-AmelX; Santa Cruz, clone FL-191, 1:200; anti-Ambn; Santa Cruz, clone M-300,1:200; anti-CD90 PE-labeled; Biolegend, clone OX-7, 1:500). After washing in PBS, samples were incubated with secondary antibodies (anti-Rabbit IgG Alexa Fluor488; 1:800; Invitrogen) for 30 min, washed, and mounted using Prolong Gold Mounting Media containing Dapi (Invitrogen, United States).

### Ca^2+^ Imaging

For cytosolic Ca^2+^concentration ([Ca^2+^]_cyt_) measurements, isolated secretory and maturation EO cells were plated on coated cover slips for 24 h after isolation, and loaded with Fura-2/AM (Molecular Probes, United States) and CD90 (BioLegend, United States) for 30 min at room temperature. Fluorescence measurements were performed every 7 s, using a Nikon Eclipse fluorescence microscope (Chiyoda, Tokyo, Japan). Cells were excited alternatively at 340 or 380 nm and emitted fluorescence intensity was recorded at 505 nm. Data acquisition was performed by using computer software (NIS Elements, United States). We first measured SOCE by monitoring changes in cytosolic Ca^2+^ upon passively depleting intracellular Ca^2+^stores with thapsigargin (1 μM, Sigma-Aldrich) to block sarco-endoplasmic reticulum Ca^2+^ pumps (SERCA). Experiments were carried out prior to, and during exposure of the cells to the Ca^2+^-free solution (see below). Re-addition of extracellular Ca^2+^allowed us to make assessments of SOCE activity in secretory and maturation EO cells.

In a separate set of experiments, and to assess the effects of physiological activators in mobilizing Ca^2+^, changes in [Ca^2+^]_cyt_ were monitored prior to and following addition of ACh, CCK and ATP to the Ringer solution. [Ca^2+^]_cyt_ was estimated from calibration curves by using the Calcium Calibration Buffer Kit#2 (Molecular Probes, Life technologies, United States) as described (Grynkiewicz et al., 1985). All experiments were performed at 25°C. Ringer solution contained (in mmol/l): 125 NaCl, 5 KCl, 1.2 MgSO_4_, 32.2 Hepes, 2 Na_2_HPO_4_, 2 CaCl_2_, and 5 glucose (pH 7.4). For the Ca^2+^-free solution, the Ringer solution was supplemented with 0.5 mM EGTA. Moreover, the effect of ATP, CCK and ACh and their ability to elicit Ca^2+^release from intracellular stores was measured following addition of acetylcholine chloride (ACh, 10 μM, Sigma, United States), adenosine 5′-triphosphate disodium salt hydrate (ATP, 100 μM, Sigma, United States) or sulfated CCK8 (CCK8S) (50 nM, 10 nM, and 10 pM, Sigma, United States) to the Ca^2+^-free solution. Secretory and maturation EO cells were also pre-treated with the CRAC channel inhibitors Synta-66 (3 μM, Aobious, United States) or GSK-7975A (10 μM, Aobious, United States) for 30 min before initiating the experiment and then Synta-66 or GSK-7975A were present throughout the experiment. This was done to ascertain their effect in modulating changes in [Ca^2+^]_cyt_ when blocking CRAC channels. Both Synta-66 and GSK-7975A are potent pharmacological blockers of ORAI1, the pore subunit of the CRAC channel (Derler et al., 2013).

### RT-PCR

Total RNA was extracted from rat secretory and maturation EO cells as described (Lacruz et al., 2012). Briefly, cell homogenates were treated using RNeasy^*R*^ Micro Kit (Qiagen, United States) according to the manufacturer’s specifications and converted to cDNA using iScript^TM^ cDNA Synthesis Kit (Bio-Rad, United States). RT-PCR amplifications were done using SsoAdvanced^TM^Universal SYBR Green Supermix (BioRad, United States) on a CFX Connect^TM^ Real-Time System (Bio-Rad, United States). Primer specificity was confirmed by analysis of a melting curve. All experiments were done in triplicates. Beta-actin was amplified to standardize the amount of sample RNA. Relative quantification of gene expression was achieved using the ΔCT method (Pfaffl;, 2001). Supplementary Table S1 lists all primer sequences used.

### *In Situ* Hybridization (ISH)

Rat Cck (NCBI accession #NM_012829.2) was amplified using probes shown in Supplementary Table S2. Rat brain cDNA was used as template. The 585 bp PCR product was ligated into pGEMTeasy (promega) using digestive enzymes Sal I (SP6) and Sac II (T7) and sequenced. This construct is referred to as pGEMT-CCK. Sense (negative control) and antisense digoxigenin (DIG)-labeled RNA probes (Genious kit; Roche Diagnostics, Indianapolis, IN, United States) were synthesized using linearized pGEMT-CCK. For *in situ* hybridization of CCK, 10-day-old rats were euthanized. Isolated mandibles were stripped of soft tissues as described above and fixed in 4% paraformaldehyde (Affymetrix USB, United States) overnight at 4°C, and decalcified in 10% EDTA (pH 7.3) for 7–10 days and washed. Brain was used as a control and processed as for dental tissues but without the decalcification step. Tissues were embedded in Paraplast ^+^ and ∼5 μm sections collected on a glass slide, deparaffinized and hydrated, and hybridized overnight at 60°C in a humidified chamber with DIG-labeled *Cck* probes. Sections were then washed in 2x SSPE (saline sodium phosphate EDTA; Invitrogen, Grand Island, NY, United States). Probes were detected using an anti-DIG antibody conjugated to alkaline phosphatase at a 1:2000 dilution for 2 h at room temperature. After several washes, the chromogenic reaction was performed overnight by incubation in BM purple (Roche, Indianapolis, IN, United States). The reaction was stopped by fixation in formalin. The sections were briefly counterstained with eosin, dehydrated and mounted in Permount (Fisher Scientific, Pittsburgh, PA, United States).

### Statistics

Data collected were sorted, coded, and entered into a Microsoft Excel spreadsheet for analysis using GraphPad Prism 5.0 (San Diego, CA, United States). Data are provided as means ± SEM, *n* represents the number of independent experiments. For the Ca^2+^ imaging experiments, a minimum of three independent experiments were performed. Each experiment consisted of pools of secretory and maturation EO cells from three rats. The number of cells used per measurement is detailed in each Figure. For RT-PCR experiments, we used a total of six rats. In all experiments, males and females were used. Cells were pooled from a pair of animals. Differences were tested for significance using 2-tailed unpaired Student’s *t*-test. *P* < 0.05 was considered statistically significant.

## Results

### SOCE Measurements in Single Ameloblasts

As Ca^2+^ requirements are higher during enamel maturation than in the preceding secretory stage, a primary requisite was to determine whether SOCE is also elevated during maturation. In a previous study, we reported increased SOCE during maturation in a mixed cell population of rat primary EO cells analyzed in a plate reader (Nurbaeva et al., 2015a). Although such mixed populations are largely composed of ameloblasts (Chen et al., 1998), contamination by fibroblasts is likely. In the present study, we developed a protocol to obtain single-cell [Ca^2+^]_cyt_ recordings that enabled us to discriminate ameloblasts by using the fibroblast marker CD90 to eliminate these cells. The overwhelming predominance of ameloblasts was further validated histologically using two marker proteins, amelogenin and ameloblastin (Supplementary Figure S1).

**FIGURE 1:**
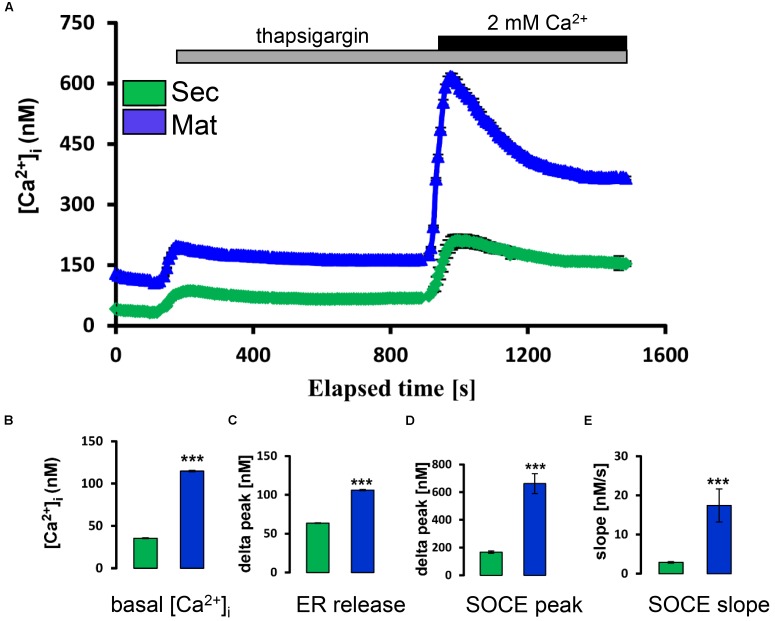
Store-operated Ca^2+^ entry in ameloblasts. **(A)** Ca^2+^ imaging ([Ca^2+^]_cyt_) of individual secretory and maturation ameloblasts (*green* and *blue* tracings, respectively). Where indicated, thapsigargin (TG, 1 μM) was added to the Ca^2+^-free bath solution to monitor the release of Ca^2+^ from ER/Ca^2+^ stores. Increased [Ca^2+^]_cyt_ upon re-addition of extracellular Ca^2+^ (2mM Ca^2+^) in the continued presence of TG reflects SOCE. **(B)** Aggregate data for basal [Ca^2+^]_cyt_ of secretory and maturation ameloblasts (before addition of thapsigargin). **(C)** Aggregate data for peak [Ca^2+^]_cyt_ reached after initial exposure to thapsigargin (ER release, at about 200 s). **(D,E)** Peak and subsequent slope of decay for [Ca^2+^]_cyt_ following re-addition of 2 mM in the ongoing presence of thapsigargin. All aggregate data **(B–E)** are means ± SEM, with *n* = 210 cells (secretory) and *n* = 163 cells (maturation). ^∗∗∗^*P* < 0.001, 2-tailed unpaired Student’s *t*-test.

Secretory and maturation stage ameloblasts were loaded with Fura-2 as reported (Nurbaeva et al., 2015a). We found that secretory ameloblasts showed lower basal [Ca^2+^]_cyt_ than those at maturation stage (**Figures 1A,B**). Stimulation of each cell type with thapsigargin to passively deplete the ER/Ca^2+^ stores revealed higher Ca^2+^ release from maturation cells (**Figure 1C**). The re-addition of Ca^2+^ to the extracellular medium to stimulate SOCE elicited significantly higher SOCE in maturation ameloblasts (**Figure 1D**), validating our previous findings from the mixed cell populations (Nurbaeva et al., 2015a).

### Molecular Analysis of Candidate SOCE Regulators

**FIGURE 2:**
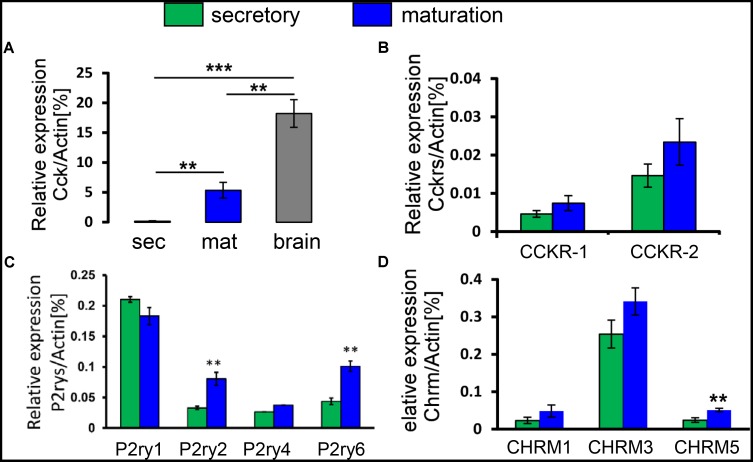
Transcript profiling of CCK and GPCRs for CCK, ACh, and ATP in enamel cells. **(A)** Relative expression levels of the *Cck* gene in secretory and maturation enamel cells versus brain, as determined by RT-PCR. *Cck* mRNA was significantly higher during maturation than secretory stage, and reached about a third of the expression level in brain. **(B)** Relative expression levels of the *CCKR-1* and *CCKR-2* genes, showing a predominance of *CCKR-2* and a trend toward upregulation during maturation. **(C)** Relative expression levels of purinergic ATP receptor genes, showing detection of *P2ry1*, *P2ry2*, *P2ry4*, and *P2ry6*. Both *P2ry2* and *P2ry6* were significantly upregulated during maturation. **(D)** Relative expression levels of ACh receptors, showing detection of *CHRM1*, *CHRM3*, and *CHRM5* and with *CHRM5* being significantly upregulated during maturation. Data (mean ± SEM) were normalized using Actin as a reference gene in all experiments. ^∗∗^*P* < 0.01; ^∗∗∗^*P* < 0.001, 2-tailed unpaired Student’s *t*-test. Values represent a minimum of three independent experiments each pooling cells from two rats per experiment.

Our previous genome-wide analysis of enamel cells revealed a ∼200-fold upregulation of *Cck* during maturation (Lacruz et al., 2012). Recognition that CCK is an important agonist that can stimulate a rise in [Ca^2+^]_cyt_ in pancreatic acinar cells (Yule et al., 1991; Yule and Williams, 1994) led us to consider a potential role of CCK in Ca^2+^ handling in enamel cells. To address this possibility, we first extended our previous microarray data by performing RT-PCR. We found a significant upregulation of *Cck* during maturation, giving an expression level which was about a third of that in brain, a CCK-enriched tissue (**Figure 2A**). To reinforce these gene expression findings, we analyzed maturation-stage ameloblasts for *Cck* expression by *in situ* hybridization (Supplementary Figure S2). Using brain as a positive control, CCK was found to be expressed by maturation ameloblasts but not by surrounding cells of the EO (Supplementary Figure S2). These findings led us to consider whether ameloblasts would respond to CCK stimulation via its GPCRs, CCKR-1 and CCKR-2 (Wank, 1998). RT-PCR showed that both receptor types were expressed in enamel cells, with *CCKR-2* predominating about threefold over *CCKR-1*. Moreover, both *CCKR-1* and *CCKR-2* were upregulated during maturation (**Figure 2B**). We also investigated the expression of gastrin, a homolog of CCK that can also bind to CCKRs. However, no gastrin transcripts were detected by RT-PCR in enamel cells (data not shown).

Two other well-known physiological agonists, acetylcholine (ACh) and ATP, can also stimulate a rise in [Ca^2+^]_cyt_ acting via their respective cell surface receptors. Muscarinic ACh receptors (CHRM) are GPCRs with the CHRM1, CHRM3, and CHRM5 subtypes being linked to IP_3_ production and a consequent rise in [Ca^2+^]_cyt_ (Caulfield, 1993; Horn et al., 2000). Using RT-PCR for *CHRM1*, *CHRM3*, and *CHRM5*, we found that *CHRM3* expression levels were the highest although no significant differences were detected between the secretory and maturation stages (**Figure 2C**). ATP is known as an intracellular energy source that also functions as an extracellular messenger by binding to purinergic cell surface receptors (Burnstock, 1997). In rat, *P2ry1*, *P2ry2*, *P2ry4*, and *P2ry6* are the GPCRs whose activation elevates [Ca^2+^]_cyt_ (Haanes and Edvinsson, 2014). Secretory and maturation cells expressed all 4 of these ATP receptors with a significant upregulation of *P2ry2* and *P2ry6* during maturation (**Figure 2D**). Collectively, these results suggest that CCK, ACh, and ATP might contribute to Ca^2+^ handling in enamel cells, possibly via regulation of SOCE.

### Functional Evidence of SOCE Regulation by CCK, ACh, and ATP in Enamel Cells

Following our observation that ameloblasts express CCK and its receptors as well as the receptors for ACh and ATP, we next asked whether these agonists participate in SOCE in enamel cells and if they do so by modulating the CRAC channel.

**FIGURE 3:**
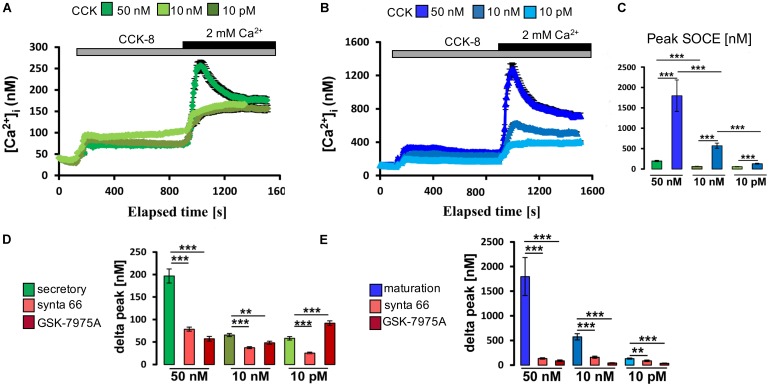
Cholecystokinin stimulates CRAC channel-mediated SOCE in ameloblasts. **(A)** Representative individual cell tracings for secretory ameloblasts exposed to various concentrations of CCK8S in the absence and presence of extracellular Ca^2+^ as indicated. CCK8S elicited ER/Ca^2+^ release and SOCE. **(B)** Equivalent experiment to A but using maturation ameloblasts, showing higher responses for ER/Ca^2+^ release and SOCE. **(C)** Aggregate data (mean ± SEM) for SOCE peak values, showing stronger responses for maturation ameloblasts at all concentrations of CCK8S (50 nM, 10 nM, and 10 pM). Data were aggregated from multiple traces as follows: secretion ameloblasts, *n* = 120, 95, and 73 cells for 50 nM, 10 nM, and 10 pM CCK8S, respectively; maturation ameloblasts, *n* = 56, 62, and 64 cells for 50 nM, 10 nM, and 10 pM CCK8S, respectively. **(D)** Aggregate data for secretory ameloblasts exposed to CRAC channel blockers (3 μM Synta-66, 10 μM GSK-7975A) during CCK8S-induced SOCE as indicated. Both channel blockers provoked a substantial reduction in SOCE peak values (delta peak) at 50 nM CCK8S, consistent with CRAC channel-mediated SOCE. Data were aggregated as follows: controls, *n* = 120, 95, and 73 cells; Synta-66, *n* = 92, 64, and 54 cells; GSK-7975A, *n* = 37, 45, and 30 cells for 50 nM, 10 nM, and 10 pM CCK8S, respectively. **(E)** Equivalent experiment to **D** but using maturation ameloblasts, showing relatively strong responses to both channel blockers at all concentrations of CCK8S. Controls, *n* = 56, 62, and 64 cells; Synta-66, *n* = 56, 51, and 47 cells; GSK-7975A, *n* = 38, 33, and 36 for 50 nM, 10 nM, and 10 pM CCK8S, respectively. ^∗∗^*P* < 0.01, ^∗∗∗^*P* < 0.001. 2-tailed unpaired Student’s *t*-test.

CCK8 comprises the 8 C-terminal amino acids of CCK and is one of its most biologically potent forms (Liddle, 1997). CCK8 exists in sulfated and unsulfated molecular forms with the former (CCK8S) being frequently used in research studies (Brennan et al., 2008). Secretory and maturation stage ameloblasts were loaded with Fura-2 as above and stimulated with various concentrations of CCK8S (50 nM, 10 nM, or 10 pM). In the absence of extracellular Ca^2+^, stimulation with CCK8S led to a transient increase in [Ca^2+^]_cyt_ representing ER/Ca^2+^ release (**Figures 3A,B**). Re-addition of extracellular Ca^2+^ resulted in a rapid elevation in [Ca^2+^]_cyt_ via activation of SOCE, which was significantly higher in maturation (**Figure 3C**). These effects of CCK8S on SOCE were concentration-dependent at both developmental stages. To further assess if the changes elicited by CCK were directly associated with SOCE and not with other calcium pathways, we repeated these experiments using two pharmacological inhibitors, Synta-66 and GSK-7975A, to block the CRAC channels (Putney, 2010; Derler et al., 2013; Gerasimenko et al., 2013). Both inhibitors were found to block the effects of CCK8S on [Ca^2+^]_cyt_ (**Figures 3D,E**). These results indicate that SOCE mediated by CRAC channels is responsive to CCK in ameloblasts.

**FIGURE 4:**
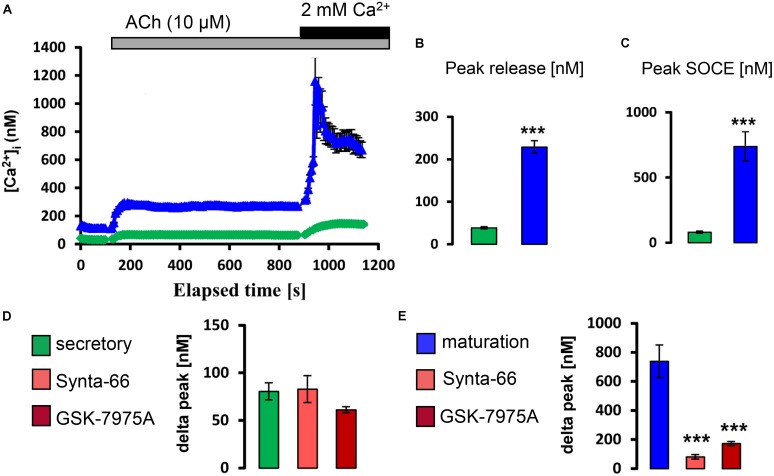
Acetylcholine stimulates CRAC channel-mediated SOCE in ameloblasts. **(A)** Representative individual cell tracings for secretory (green) and maturation (blue) ameloblasts exposed to 10 μM ACh in the absence and presence of extracellular Ca^2+^ as indicated. ACh can be seen to trigger relatively strong responses for ER/Ca^2+^ release and SOCE in maturation ameloblasts. **(B,C)** Aggregate data for peak ER/Ca^2+^ release and SOCE value for secretory (*n* = 89) and maturation ameloblasts (*n* = 64) as indicated, confirming the relatively strong responses during maturation. **(D,E)** Aggregate data for secretory and maturation ameloblasts exposed to CRAC channel blockers during ACh-induced SOCE as indicated. Both Synta-66 (3 μM) and GSK-7975A (10 μM) provoked a substantial reduction in SOCE peak values in maturation cells, consistent with CRAC channel-mediated SOCE, whereas secretory cells were unresponsive. Data were aggregated as follows: Synta-66, *n* = 54 and 37 cells; GSK-7975A, *n* = 54 and 50 cells for secretion and maturation, respectively. ^∗∗∗^*P* < 0.001, 2-tailed unpaired Student’s *t*-test.

**FIGURE 5:**
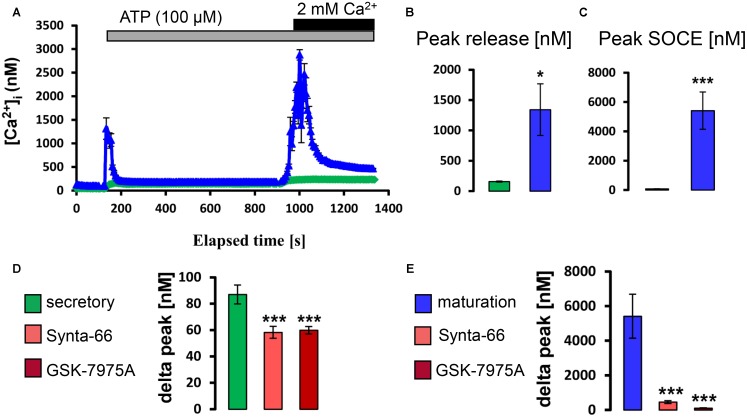
ATP stimulates CRAC channel-mediated SOCE in ameloblasts. **(A)** Representative individual cell tracings for secretory (green) and maturation (blue) ameloblasts exposed to 100 μM ATP in the absence and presence of extracellular Ca^2+^ as indicated. ATP can be seen to trigger relatively strong responses for ER/Ca^2+^ release and SOCE in maturation ameloblasts. **(B,C)** Aggregate data for peak ER/Ca^2+^ release and SOCE value for secretory (*n* = 60) and maturation ameloblasts (*n* = 82) as indicated, confirming the relatively strong responses during maturation. **(D,E)** Aggregate data for secretory and maturation ameloblasts exposed to CRAC channel blockers during ATP-induced SOCE as indicated. Both Synta-66 (3 μM) and GSK-7975A (10 μM) provoked a substantial reduction in SOCE peak values in maturation cells, consistent with CRAC channel-mediated SOCE, whereas secretory cells were much less responsive. Data were aggregated as follows: Synta-66, *n* = 55 and 40 cells; GSK-7975A, *n* = 55 and 40 cells, for secretion and maturation, respectively. ^∗∗∗^*P* < 0.001, 2-tailed unpaired Student’s *t*-test. ^∗^*P* < 0.05, ^∗∗∗^*P* < 0.001, 2-tailed unpaired Student’s *t*-test.

Next we similarly tested whether exposure to physiological levels of ACh and ATP could stimulate a rise in [Ca^2+^]_cyt_. It was found that, in the presence of external Ca^2+^, the application of ATP (100 μM) and ACh (10 μM) elevated [Ca^2+^]_cyt_ with higher increase in maturation (Supplementary Figure S3). To determine if these changes reflected SOCE, ameloblasts were exposed to ACh and to ATP in Ca^2+^ -free media before re-addition of Ca^2+^. Both ACh and ATP elicited ER/Ca^2+^ release followed by a significant elevation in [Ca^2+^]_cyt_ which was significantly higher in maturation ameloblasts (**Figures 4, 5**). Addition of Synta-66 and GSK-7975A resulted in a significant reduction of Ca^2+^ influx suggesting that the effects of ACh and ATP on [Ca^2+^]_cyt_ were mediated by CRAC channels. Together with those obtained with CCK, these results provide the first evidence of a SOCE-regulatory system that is overexpressed in maturation stage ameloblasts.

## Discussion

The mechanisms that allow ameloblasts to handle bulk Ca^2+^ safely while producing the most highly calcified tissue remain unclear. Contradicting the cytosol-based dogma held for other Ca^2+^-transporting tissues, our previous findings suggested that Ca^2+^ transits ameloblasts by an ER-based route. Here, we build on evidence suggesting that SOCE, an ER-linked Ca^2+^ entry mechanism, plays a central role by querying the existence of a SOCE-regulatory system. We show that three physiological agonists of SOCE (CCK, ACh, and ATP) modulate Ca^2+^ entry into ameloblasts, and that this agonist-responsive SOCE is upregulated during enamel maturation in parallel with Ca^2+^-transport demand. These findings implicate enamel cells as a novel physiological target of CCK, raising the possibility of an auto/paracrine system for regulating Ca^2+^ transport.

The identification of molecular and functional differences between the secretory and maturation stages of enamel formation is an important step toward understanding the physiological requirements of these cells. Here, we have developed a protocol that enabled us to obtain single cell Ca^2+^ recordings directly from ameloblasts, the main cell type involved in handling bulk Ca^2+^ (**Figure 1**). Using this refined approach, we confirmed that maturation ameloblasts have higher SOCE and ER/Ca^2+^ store capacity relative to those at secretory stage. These findings agree with reports that mutations in the CRAC channel genes *STIM1* and *ORAI1* result in hypomineralized enamel (McCarl et al., 2009; Wang et al., 2014). All isoforms of ORAI1 and STIM1 are up-regulated during maturation, consistent with increased SOCE activity relating to the bulk transport of Ca^2+^ (Nurbaeva et al., 2015a). However, no data on potential CRAC channel activators in ameloblasts was available.

Ca^2+^ release activated Ca^2+^ channels are activated by binding of agonists to GPCRs, leading to IP_3_ production and consequent release of Ca^2+^ from ER/Ca^2+^ stores which in turn triggers SOCE (Yule et al., 1991; Cancela, 2001). *In vitro*, SOCE can be activated by blocking the ER/Ca^2+^ pump (SERCA) with thapsigargin. However, thapsigargin does not act on the IP_3_-axis and so the present study aimed to characterize the effects of physiological SOCE activators in ameloblasts. To do so, we undertook molecular characterization of cell-surface receptors (GPCRs) for three candidate agonists of SOCE (CCK, ACh, and ATP). Our transcript-profiling results (**Figure 2**) revealed the expression of GPCRs for all three agonists, raising the possibility that they activate SOCE in ameloblasts.

The peptide hormone CCK is mostly synthesized in the brain but cells of the small intestine also produce it (Rehfeld, 1978; Larsson, 1980). Here, we identified *Cck* expression in ameloblasts for the first time by using RT-PCR and *in situ* hybridization (**Figure 2** and Supplementary Figure S2). Notably, *Cck* transcript levels were substantially higher in maturation cells than during secretion, reaching a relatively high expression level (about a third of that in brain) that suggests physiological significance (**Figure 2D**). The finding that this peptide hormone was so highly expressed in Ca^2+^-transporting ameloblasts is surprising. Interestingly, CCK is known to function as an autocrine hormone (Carrillo et al., 2007). For CCK to be a locally acting hormone it needs to be synthesized, secreted and released in the vicinity of target cells. Despite our evidence that CCK is synthesized by maturation ameloblasts, we could not detect protein expression by Western blot analysis using CCK8 antibodies. Thus whether CCK acts locally on maturation ameloblasts or other cells in the EO cannot be directly addressed at this stage. However, the new prospect that CCK might comprise part of an auto-regulatory system for Ca^2+^ transport is appealing, particularly given CCK is involved in Ca^2+^ mobilization for cell signaling (Yule et al., 1991; Cancela, 2001).

The functional analyses with CCK endorsed our molecular evidence for its involvement in ameloblast Ca^2+^ handling, which leads us to suggest that developing enamel may be a new target tissue for CCK regulation. CCK8S stimulated the release of IP_3_-sensitive Ca^2+^ pools and SOCE, with maturation cells reacting more strongly than those at secretory stage (**Figure 3**). At physiologically low concentrations (10 pM), CCKS8 is known to activate CCKR-1 whereas CCKR-2 is activated at higher (nM) concentrations (Rehfeld, 2006). Our evidence that *CCKR-2* predominates in both secretory and maturation cells (**Figure 2B**) coupled with the increased Ca^2+^ mobilization at supra-physiological CCK concentrations (**Figure 3**) suggests that Ca^2+^ elevations in enamel cells may be modulated via CCKR-2 although this remains to be tested. Moreover, our results show that CCK stimulation can be blocked by the CRAC channel inhibitors Synta-66 and GSK-7975A, implying that the [Ca^2+^]_cyt_ increase triggered by CCK is mediated via the CRAC channel. The collective evidence that CCK modulates bulk Ca^2+^ entry into enamel cells via CRAC channels introduces the EO as a potential target of CCK regulation. Although no dental defects were reported in *Cck*-deficient mice (Lo et al., 2008), it appears that detailed dental phenotyping remains to be done in this and other pertinent genetic models.

Amplifying the conclusions about regulation by CCK, we also found that both ACh and ATP elicited CRAC channel-mediated Ca^2+^ entry into ameloblasts (**Figures 4, 5**). ACh acts via specific muscarinic GPCRs (*Chrm* gene products) to elevate [Ca^2+^]_cyt_ in many cell types (Streb et al., 1983) and we established that ameloblasts express three such *Chrm* genes (**Figure 2**). Similarly, the purinergic class of GPCRs responsible for ATP-induced [Ca^2+^]_cyt_ increase (Haanes and Edvinsson, 2014) was found to be represented by *P2ry1*, *P2ry2*, *P2ry4*, and *P2ry6* (**Figure 2**). Again, although no dental defects have been reported to date in *P2ry2* and *P2yr6* knockout mice (Bar et al., 2008), detailed dental phenotyping now seems worthwhile.

Enamel mineralization requires a major supply of Ca^2+^ particularly during the cellular switch from secretion to maturation when the rate of Ca^2+^ transport increases about fourfold (Smith, 1998; Hubbard, 2000). In ameloblasts, which are elongate and highly polarized epithelial cells, enamel forms at the apical/distal pole far away (≈ 80 μm) from the blood vessels that supply the necessary ions for mineralization. Our previous work pointed to an organellar route that directs Ca^2+^ safely across ameloblasts prior to extrusion largely by the sodium/calcium/potassium exchanger, NCKX4 (Hu et al., 2012; Lacruz et al., 2013). This ’Ca^2+^ transcytosis’ paradigm requires a developmentally regulated Ca^2+^-entry step to maintain Ca^2+^ levels within the organellar Ca^2+^ stores (e.g., ER and mitochondria). Data presented here and in our previous studies (Nurbaeva et al., 2015a; Eckstein et al., 2017) firmly establish SOCE via CRAC channels as a key mechanism for Ca^2+^ entry into maturation ameloblasts. The SOCE upregulation in maturation points to a system that modulates SOCE activity to accommodate Ca^2+^-signaling and Ca^2+^-transport needs. Here, we have shown that the physiological agonists CCK, ACh and ATP all stimulate CRAC channel mediated Ca^2+^ entry in ameloblasts, thus providing the first evidence of such a SOCE-regulatory system in enamel development.

## Conclusion

Molecular and functional data presented here show that ameloblasts can be stimulated by the physiological agonists CCK, ACh, and ATP to elicit elevations in [Ca^2+^]_cyt_. This provides the first evidence of a regulatory system for SOCE, a Ca^2+^ entry mechanism linked to the ER. Moreover, we showed significantly higher capacity for SOCE in maturation ameloblasts than secretory cells. This study is also the first to identify enamel cells as a target of the peptide hormone CCK, raising the possibility of an autocrine/paracrine role of CCK in enamel formation. Additionally, CCK, ACh, and ATP are positive modulators of CRAC channel-mediated Ca^2+^ influx in ameloblasts, as evidenced by sensitivity to pharmacological inhibitors. Taken together, these findings contribute to a better understanding of enamel cell physiology in particular the regulation of Ca^2+^ homeostasis.

## Author Contributions

MN and RL designed the project. MN, ME, and AD collected the data. MN, ME, J-PS-J, MH, DY, and RL analyzed the data. MN, ME, DY, MH, and RL wrote the paper.

## Conflict of Interest Statement

The authors declare that the research was conducted in the absence of any commercial or financial relationships that could be construed as a potential conflict of interest.
