# Supplementary material for: Evidence That Calcium Entry Into Calcium-Transporting Dental Enamel Cells Is Regulated by Cholecystokinin, Acetylcholine and ATP
Source: Front Physiol. 2018 Jul 2;9:801. doi: 10.3389/fphys.2018.00801 (PMC6036146; doi:10.3389/fphys.2018.00801)
Supplement: Supplementary file 1 [file Data_Sheet_1.PDF]

Supplementary Information:

Title: Evidence that calcium entry into calcium-transporting dental enamel cells is regulated by cholecystokinin, acetylcholine and ATP.

Authors: Meerim K. Nurbaeva[1], Miriam Eckstein[1], Arun Devotta[1], Jean-Pierre Saint Jeannette[1], David I. Yule[2], Michael J. Hubbard[3], Rodrigo S. Lacruz[1]

[1]Dept. of Basic Science and Craniofacial Biology, New York University College of Dentistry, New York, NY, USA

[2]Dept. Pharmacology and Physiology, University of Rochester, Rochester NY, USA.

[3]Depts. of Paediatrics and Pharmacology, The University of Melbourne, Parkville, Victoria 3010, Australia.

Address for correspondence:

Rodrigo S. Lacruz
NYU College of Dentistry
345 East 24th Street, room #1007
New York, 10010NY
Phone: 212 998 9433
Fax: 212 995 4087
E-mail: rodrigo.lacruz@nyu.edu

Figure S1. Purity assessment of isolated enamel cells in primary culture.

A) Light microscopy showing cells isolated from enamel epithelium at secretion stage and stained with anti-CD90 to reveal fibroblast contamination. Fibroblasts were readily detected as sparse, making it easy to avoid them during the Ca^{2+} imaging experiments.

B) Equivalent experiment to **A** but at maturation stage. A prominent fibroblast is arrowed.

C and D) Immunofluorescence microscopy of isolated enamel cells labelled with markers for ameloblasts (green) and fibroblasts (red). Cells isolated from enamel epithelium at secretory (SEO) and maturation (MEO) stages were immunolabelled for amelogenin (AmelX), ameloblastin (Ambn) and CD90 as indicated, and nuclei were stained with DAPI (blue). Green cells (i.e. positive for ameloblast markers amelogenin and ameloblastin) predominated at both developmental stages, with only a minimal contribution of red cells (i.e. CD90-positive fibroblasts).

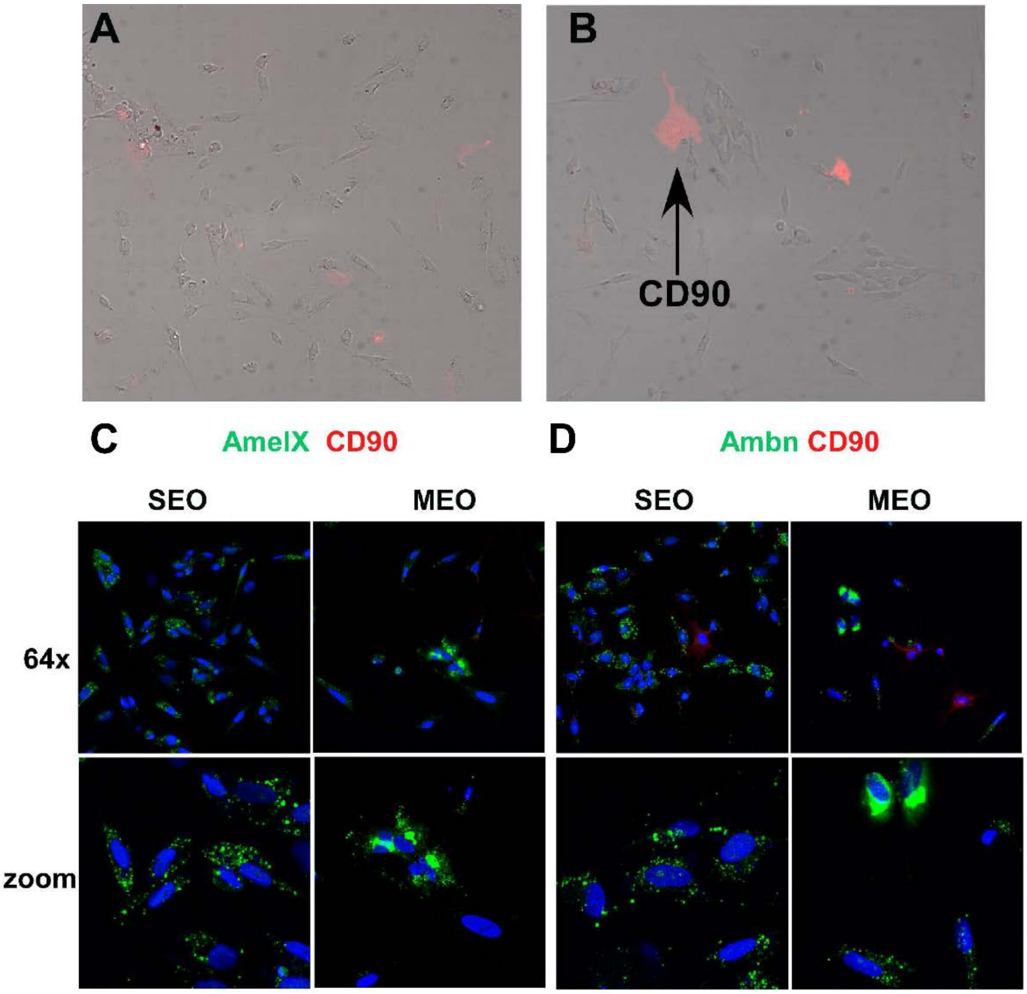

Figure S2. *In situ* hybridization shows specific expression of *CCK* mRNA in rat ameloblasts. A-B) Brain was used as a positive control to validate the *CCK* sense and anti-sense probes. Strong specific staining is evident in the hippocampus as expected.

C-D) Parallel analysis of developing rat incisor revealed strong CCK staining in maturation ameloblasts, whereas adjacent epithelial accessory cells and connective tissues were negative.

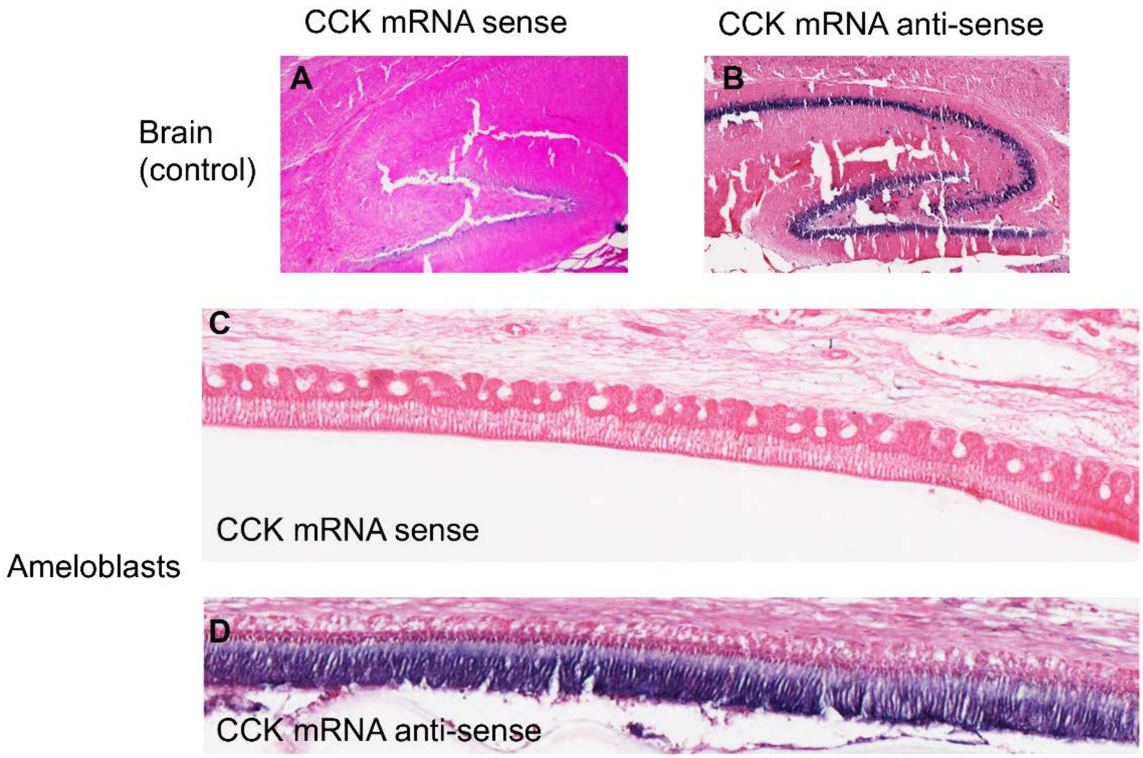

Figure S3. ATP and ACh stimulate a rise in cytosolic calcium.

A) Stimulation with ATP induced a substantial increase in $[Ca^{2+}]_{cyt}$, particularly in maturation ameloblasts. Shown here are representative tracings from secretory (green) and maturation (blue) ameloblasts before and after exposure to ATP (100 μM) in the presence of extracellular Ca^{2+}.

B) Aggregate data for Peak $[Ca^{2+}]_{cyt}$ values elicited by ATP (means ± SEM)in secretion (*n*=106 cells) and maturation (*n*=99 cells) stages.

C and D) Equivalent experiments to A and B but with exposure to 10μM ACh at secretion (n=36 cells) and maturation (n=55 cells). *** *P*<0.001, 2-tailed unpaired Student's *t*-test.

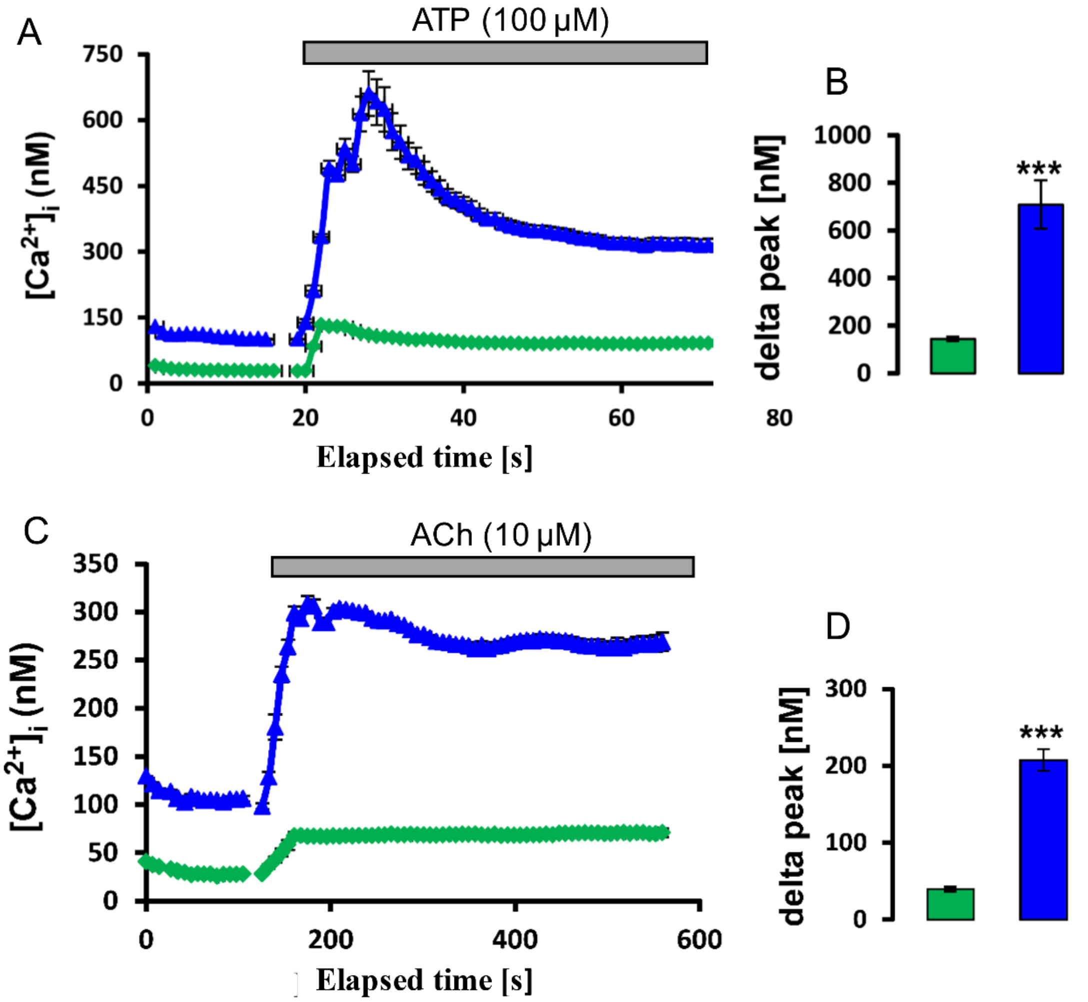

Table S1: Primers used in this study for real time PCR:

Gene name	FWD	REV
Chrm1	5'-GCA CAG GCA CCC ACC AAG CAG-3'	5'-AGA GCA GCA GCA GGC GGA ACG-3'
Chrm2	5'-CAC GAA ACC TCT GAC CTA CCC-3'	5'-TCT GAC CCG ACG ACC CAA CTA-3'
Chrm3	5'-GTC TGG CTT GGG TCA TCT CCT-3'	5'-GCT GCT GCTGTG GTC TTG GTC-3'
Chrm4	5'-TAC TTC TGC GTC ACC AAA CC- 3'	5'-CCA GAG CAC AAA GGA CAA GA- 3'
Chrm5	5'-CTG GTC TCC TTC ATC CTCTGG- 3'	5'-CCT GGG TTG TCT TTC CTG TTG-3'
P2ry1	5'- ATG TCA GTGTGCTGGTAT GG -3'	5'- GTC GTA GCA GGT GAC AGT TT -3'
P2ry2	5'- GGA CCT AAA GAG GAA CGA ACA C -3'	5'- TCG GGA CAG AGT CAC GTA AT -3'
P2ry3	5'- CTG GGT GTTTGGTTG GTA GT -3'	5'- CAT GGC ACA GGATGGTAGTT -3'
P2ry6	5'- CCT GTT CCT CAC CTG CAT TAG -3'	5'- AGC CAA ACG ACT CCA CAT AC -3'
Cck	5'- GTCCCTGTAGAAGCTGTGGA-3'	5'- ATCCTATGGCTAGGGTCCAG-3'
Cck-ar	5'- ATCTTGCACTGACCCCATTA-3'	5'- AATGAAGTCAAACGGGAAC-3'
Cck-br	5'- GCTGATTCGAAACAAGAGGA-3'	5'- TCCAGAGAGATGGCTACCAG-3'

Table S 2. Primers used for in-situ hybridization of CCK

Sequences based on the rat Cck gene (NCBI accession #NM_012829.2)

Sense: GACTCCGCATCCGAAGATATG

Anti-sense: TAGCATAGCAACATTAGGTCTGG
